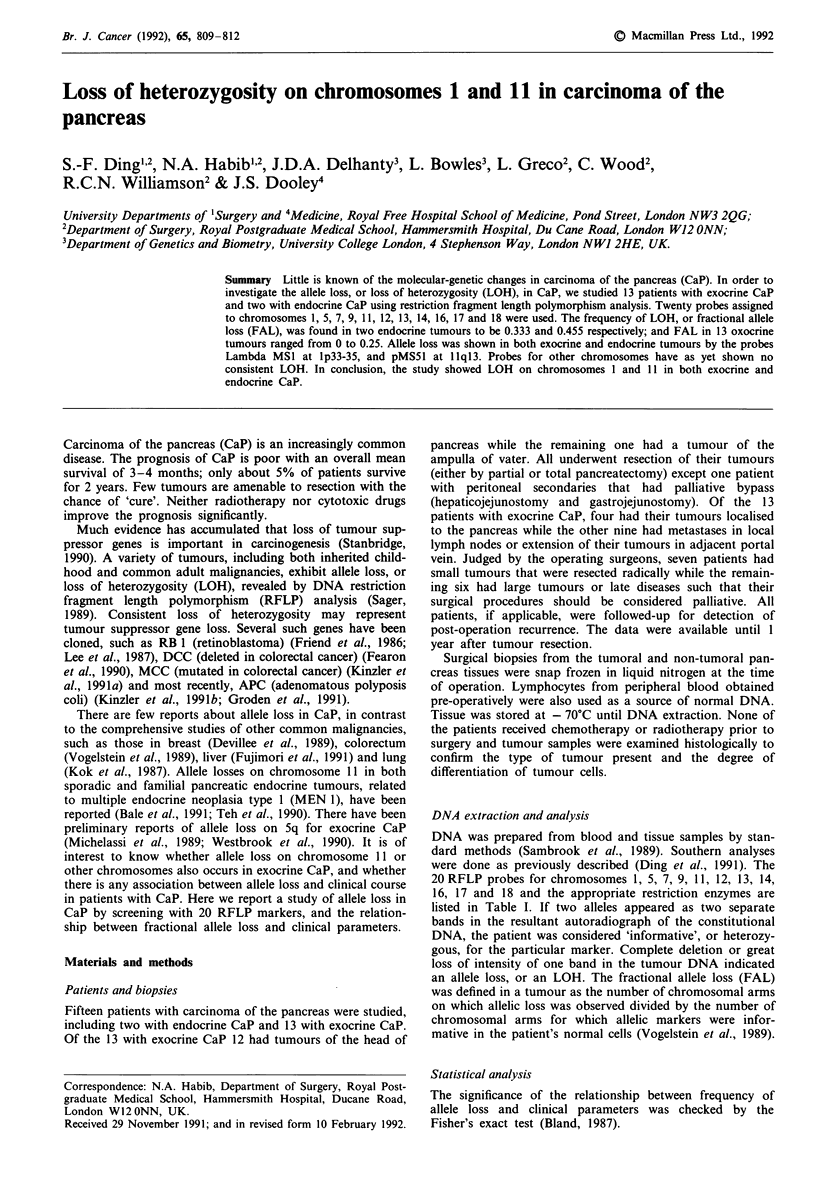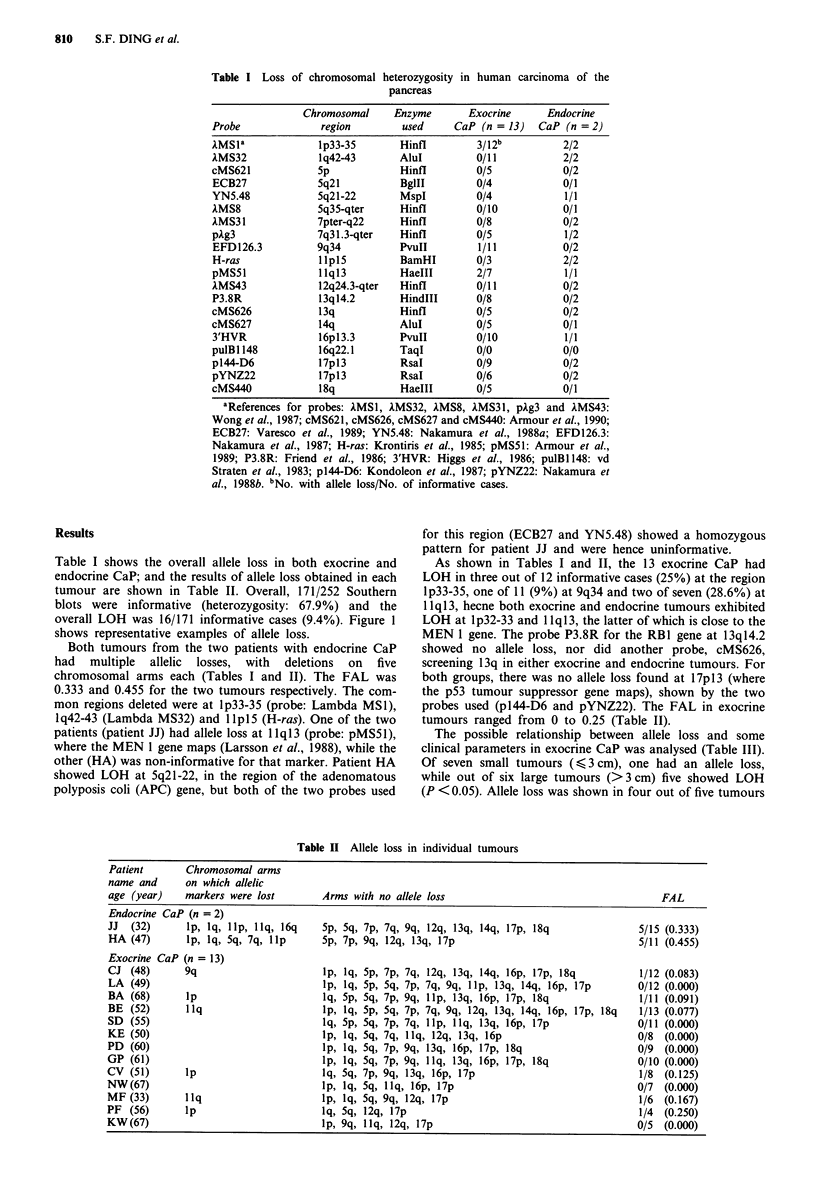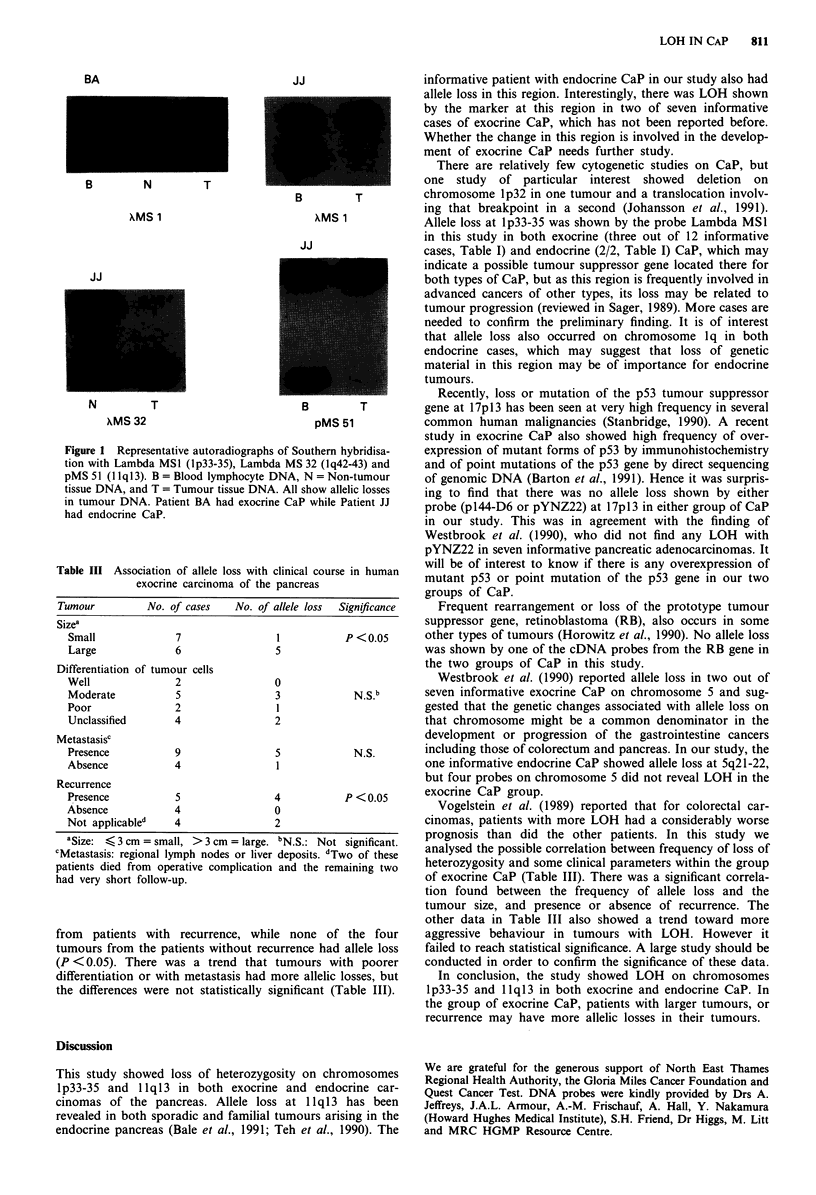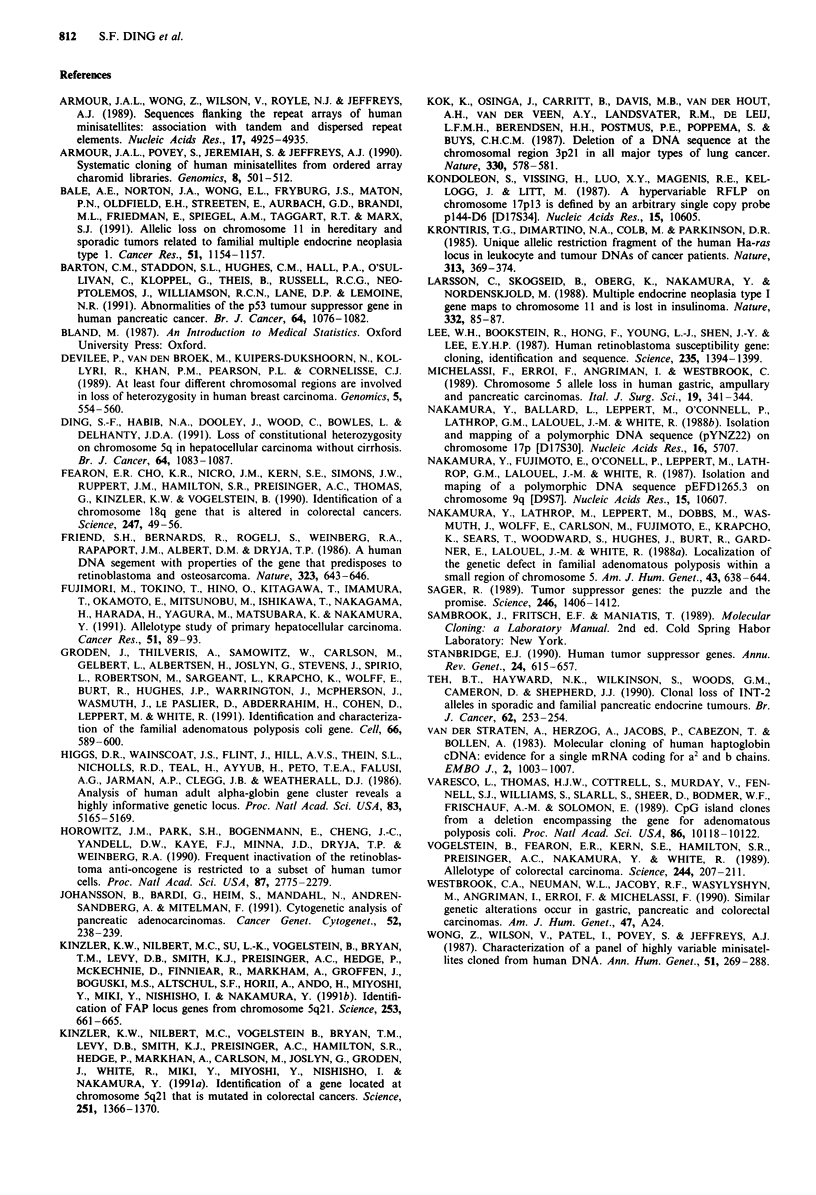# Loss of heterozygosity on chromosomes 1 and 11 in carcinoma of the pancreas.

**DOI:** 10.1038/bjc.1992.173

**Published:** 1992-06

**Authors:** S. F. Ding, N. A. Habib, J. D. Delhanty, L. Bowles, L. Greco, C. Wood, R. C. Williamson, J. S. Dooley

**Affiliations:** University Department of Surgery, Royal Free Hospital School of Medicine, London, UK.

## Abstract

**Images:**


Br. J. Cancer (1992), 65, 809-812 © Macmillan Press Ltd., 1992

### Loss of heterozygosity on chromosomes 1 and 11 in carcinoma of the pancreas

S.-F. Ding[1,2], N.A. Habib[1,2], J.D.A. Delhanty[3], L. Bowles[3], L. Greco[2], C. Wood[2], R.C.N. Williamson[2] & J.S. Dooley[4]

*University Departments of [1]Surgery and [4]Medicine, Royal Free Hospital School of Medicine, Pond Street, London NW3 2QG; [2]Department of Surgery, Royal Postgraduate Medical School, Hammersmith Hospital, Du Cane Road, London W12 0NN; [3]Department of Genetics and Biometry, University College London, 4 Stephenson Way, London NW1 2HE, UK.*

**Summary** Little is known of the molecular-genetic changes in carcinoma of the pancreas (CaP). In order to investigate the allele loss, or loss of heterozygosity (LOH), in CaP, we studied 13 patients with exocrine CaP and two with endocrine CaP using restriction fragment length polymorphism analysis. Twenty probes assigned to chromosomes 1, 5, 7, 9, 11, 12, 13, 14, 16, 17 and 18 were used. The frequency of LOH, or fractional allele loss (FAL), was found in two endocrine tumours to be 0.333 and 0.455 respectively; and FAL in 13 oxocrine tumours ranged from 0 to 0.25. Allele loss was shown in both exocrine and endocrine tumours by the probes Lambda MS1 at 1p33-35, and pMS51 at 11q13. Probes for other chromosomes have as yet shown no consistent LOH. In conclusion, the study showed LOH on chromosomes 1 and 11 in both exocrine and endocrine CaP.

Carcinoma of the pancreas (CaP) is an increasingly common disease. The prognosis of CaP is poor with an overall mean survival of 3–4 months; only about 5% of patients survive for 2 years. Few tumours are amenable to resection with the chance of 'cure'. Neither radiotherapy nor cytotoxic drugs improve the prognosis significantly.

Much evidence has accumulated that loss of tumour suppressor genes is important in carcinogenesis (Stanbridge, 1990). A variety of tumours, including both inherited childhood and common adult malignancies, exhibit allele loss, or loss of heterozygosity (LOH), revealed by DNA restriction fragment length polymorphism (RFLP) analysis (Sager, 1989). Consistent loss of heterozygosity may represent tumour suppressor gene loss. Several such genes have been cloned, such as RB 1 (retinoblastoma) (Friend *et al.*, 1986; Lee *et al.*, 1987), DCC (deleted in colorectal cancer) (Fearon *et al.*, 1990), MCC (mutated in colorectal cancer) (Kinzler *et al.*, 1991*a*) and most recently, APC (adenomatous polyposis coli) (Kinzler *et al.*, 1991*b*; Groden *et al.*, 1991).

There are few reports about allele loss in CaP, in contrast to the comprehensive studies of other common malignancies, such as those in breast (Devillee *et al.*, 1989), colorectum (Vogelstein *et al.*, 1989), liver (Fujimori *et al.*, 1991) and lung (Kok *et al.*, 1987). Allele losses on chromosome 11 in both sporadic and familial pancreatic endocrine tumours, related to multiple endocrine neoplasia type 1 (MEN 1), have been reported (Bale *et al.*, 1991; Teh *et al.*, 1990). There have been preliminary reports of allele loss on 5q for exocrine CaP (Michelassi *et al.*, 1989; Westbrook *et al.*, 1990). It is of interest to know whether allele loss on chromosome 11 or other chromosomes also occurs in exocrine CaP, and whether there is any association between allele loss and clinical course in patients with CaP. Here we report a study of allele loss in CaP by screening with 20 RFLP markers, and the relationship between fractional allele loss and clinical parameters.

### Materials and methods

### *Patients and biopsies*

Fifteen patients with carcinoma of the pancreas were studied, including two with endocrine CaP and 13 with exocrine CaP. Of the 13 with exocrine CaP 12 had tumours of the head of pancreas while the remaining one had a tumour of the ampulla of vater. All underwent resection of their tumours (either by partial or total pancreatectomy) except one patient with peritoneal secondaries that had palliative bypass (hepaticojejunostomy and gastrojejunostomy). Of the 13 patients with exocrine CaP, four had their tumours localised to the pancreas while the other nine had metastases in local lymph nodes or extension of their tumours in adjacent portal vein. Judged by the operating surgeons, seven patients had small tumours that were resected radically while the remaining six had large tumours or late diseases such that their surgical procedures should be considered palliative. All patients, if applicable, were followed-up for detection of post-operation recurrence. The data were available until 1 year after tumour resection.

Surgical biopsies from the tumoral and non-tumoral pancreas tissues were snap frozen in liquid nitrogen at the time of operation. Lymphocytes from peripheral blood obtained pre-operatively were also used as a source of normal DNA. Tissue was stored at − 70°C until DNA extraction. None of the patients received chemotherapy or radiotherapy prior to surgery and tumour samples were examined histologically to confirm the type of tumour present and the degree of differentiation of tumour cells.

### *DNA extraction and analysis*

DNA was prepared from blood and tissue samples by standard methods (Sambrook *et al.*, 1989). Southern analyses were done as previously described (Ding *et al.*, 1991). The 20 RFLP probes for chromosomes 1, 5, 7, 9, 11, 12, 13, 14, 16, 17 and 18 and the appropriate restriction enzymes are listed in Table I. If two alleles appeared as two separate bands in the resultant autoradiograph of the constitutional DNA, the patient was considered 'informative', or heterozygous, for the particular marker. Complete deletion or great loss of intensity of one band in the tumour DNA indicated an allele loss, or an LOH. The fractional allele loss (FAL) was defined in a tumour as the number of chromosomal arms on which allelic loss was observed divided by the number of chromosomal arms for which allelic markers were informative in the patient's normal cells (Vogelstein *et al.*, 1989).

### *Statistical analysis*

The significance of the relationship between frequency of allele loss and clinical parameters was checked by the Fisher's exact test (Bland, 1987).

Correspondence: N.A. Habib, Department of Surgery, Royal Postgraduate Medical School, Hammersmith Hospital, Ducane Road, London W12 0NN, UK.
Received 29 November 1991; and in revised form 10 February 1992.

Table I Loss of chromosomal heterozygosity in human carcinoma of the pancreas

| Probe | Chromosomal region | Enzyme used | Exocrine CaP (n = 13) | Endocrine CaP (n = 2) |
|---|---|---|---|---|
| λMS1[a] | 1p33-35 | HinfI | 3/12[b] | 2/2 |
| λMS32 | 1q42-43 | AluI | 0/11 | 2/2 |
| cMS621 | 5p | HinfI | 0/5 | 0/2 |
| ECB27 | 5q21 | BglII | 0/4 | 0/1 |
| YN5.48 | 5q21-22 | MspI | 0/4 | 1/1 |
| λMS8 | 5q35-qter | HinfI | 0/10 | 0/1 |
| λMS31 | 7pter-q22 | HinfI | 0/8 | 0/2 |
| pλg3 | 7q31.3-qter | HinfI | 0/5 | 1/2 |
| EFD126.3 | 9q34 | PvuII | 1/11 | 0/2 |
| H-*ras* | 11p15 | BamHI | 0/3 | 2/2 |
| pMS51 | 11q13 | HaeIII | 2/7 | 1/1 |
| λMS43 | 12q24.3-qter | HinfI | 0/11 | 0/2 |
| P3.8R | 13q14.2 | HindIII | 0/8 | 0/2 |
| cMS626 | 13q | HinfI | 0/5 | 0/2 |
| cMS627 | 14q | AluI | 0/5 | 0/1 |
| 3'HVR | 16p13.3 | PvuII | 0/10 | 1/1 |
| pulB1148 | 16q22.1 | TaqI | 0/0 | 0/0 |
| p144-D6 | 17p13 | RsaI | 0/9 | 0/2 |
| pYNZ22 | 17p13 | RsaI | 0/6 | 0/2 |
| cMS440 | 18q | HaeIII | 0/5 | 0/1 |

[a]References for probes: λMS1, λMS32, λMS8, λMS31, pλg3 and λMS43: Wong *et al.*, 1987; cMS621, cMS626, cMS627 and cMS440: Armour *et al.*, 1990; ECB27: Varesco *et al.*, 1989; YN5.48: Nakamura *et al.*, 1988*a*; EFD126.3: Nakamura *et al.*, 1987; H-*ras*: Krontiris *et al.*, 1985; pMS51: Armour *et al.*, 1989; P3.8R: Friend *et al.*, 1986; 3'HVR: Higgs *et al.*, 1986; pulB1148: vd Straten *et al.*, 1983; p144-D6: Kondoleon *et al.*, 1987; pYNZ22: Nakamura *et al.*, 1988*b*. [b]No. with allele loss/No. of informative cases.

### Results

Table I shows the overall allele loss in both exocrine and endocrine CaP; and the results of allele loss obtained in each tumour are shown in Table II. Overall, 171/252 Southern blots were informative (heterozygosity: 67.9%) and the overall LOH was 16/171 informative cases (9.4%). Figure 1 shows representative examples of allele loss.

Both tumours from the two patients with endocrine CaP had multiple allelic losses, with deletions on five chromosomal arms each (Tables I and II). The FAL was 0.333 and 0.455 for the two tumours respectively. The common regions deleted were at 1p33-35 (probe: Lambda MS1), 1q42-43 (Lambda MS32) and 11p15 (H-*ras*). One of the two patients (patient JJ) had allele loss at 11q13 (probe: pMS51), where the MEN 1 gene maps (Larsson *et al.*, 1988), while the other (HA) was non-informative for that marker. Patient HA showed LOH at 5q21-22, in the region of the adenomatous polyposis coli (APC) gene, but both of the two probes used for this region (ECB27 and YN5.48) showed a homozygous pattern for patient JJ and were hence uninformative.

As shown in Tables I and II, the 13 exocrine CaP had LOH in three out of 12 informative cases (25%) at the region 1p33-35, one of 11 (9%) at 9q34 and two of seven (28.6%) at 11q13, hecne both exocrine and endocrine tumours exhibited LOH at 1p32-33 and 11q13, the latter of which is close to the MEN 1 gene. The probe P3.8R for the RB1 gene at 13q14.2 showed no allele loss, nor did another probe, cMS626, screening 13q in either exocrine and endocrine tumours. For both groups, there was no allele loss found at 17p13 (where the p53 tumour suppressor gene maps), shown by the two probes used (p144-D6 and pYNZ22). The FAL in exocrine tumours ranged from 0 to 0.25 (Table II).

The possible relationship between allele loss and some clinical parameters in exocrine CaP was analysed (Table III). Of seven small tumours ($\leqslant 3$ cm), one had an allele loss, while out of six large tumours ($>3$ cm) five showed LOH ($P < 0.05$). Allele loss was shown in four out of five tumours

Table II Allele loss in individual tumours

| Patient name and age (year) | Chromosomal arms on which allelic markers were lost | Arms with no allele loss | FAL |
|---|---|---|---|
| *Endocrine CaP* (*n* = 2) | | | |
| JJ (32) | 1p, 1q, 11p, 11q, 16q | 5p, 5q, 7p, 7q, 9q, 12q, 13q, 14q, 17p, 18q | 5/15 (0.333) |
| HA (47) | 1p, 1q, 5q, 7q, 11p | 5p, 7p, 9q, 12q, 13q, 17p | 5/11 (0.455) |
| *Exocrine CaP* (*n* = 13) | | | |
| CJ (48) | 9q | 1p, 1q, 5p, 7p, 7q, 12q, 13q, 14q, 16p, 17p, 18q | 1/12 (0.083) |
| LA (49) | | 1p, 1q, 5p, 5q, 7p, 7q, 9q, 11p, 13q, 14q, 16p, 17p | 0/12 (0.000) |
| BA (68) | 1p | 1q, 5p, 5q, 7p, 9q, 11p, 13q, 16p, 17p, 18q | 1/11 (0.091) |
| BE (52) | 11q | 1p, 1q, 5p, 5q, 7p, 7q, 9q, 12q, 13q, 14q, 16p, 17p, 18q | 1/13 (0.077) |
| SD (55) | | 1q, 5p, 5q, 7p, 7q, 11p, 11q, 13q, 16p, 17p | 0/11 (0.000) |
| KE (50) | | 1p, 1q, 5q, 7q, 11q, 12q, 13q, 16p | 0/8 (0.000) |
| PD (60) | | 1p, 1q, 5q, 7p, 9q, 13q, 16p, 17p, 18q | 0/9 (0.000) |
| GP (61) | | 1p, 1q, 5q, 7p, 9q, 11q, 13q, 16p, 17p, 18q | 0/10 (0.000) |
| CV (51) | 1p | 1q, 5q, 7p, 9q, 13q, 16p, 17p | 1/8 (0.125) |
| NW (67) | | 1p, 1q, 5q, 11q, 16p, 17p | 0/7 (0.000) |
| MF (33) | 11q | 1p, 1q, 5q, 9q, 12q, 17p | 1/6 (0.167) |
| PF (56) | 1p | 1q, 5q, 12q, 17p | 1/4 (0.250) |
| KW (67) | | 1p, 9q, 11q, 12q, 17p | 0/5 (0.000) |

**Figure 1** Representative autoradiographs of Southern hybridisation with Lambda MS1 (1p33-35), Lambda MS 32 (1q42-43) and pMS 51 (11q13). B = Blood lymphocyte DNA, N = Non-tumour tissue DNA, and T = Tumour tissue DNA. All show allelic losses in tumour DNA. Patient BA had exocrine CaP while Patient JJ had endocrine CaP.

**Table III** Association of allele loss with clinical course in human exocrine carcinoma of the pancreas

| *Tumour* | *No. of cases* | *No. of allele loss* | *Significance* |
|---|---|---|---|
| Size[a] | | | |
| Small | 7 | 1 | $P < 0.05$ |
| Large | 6 | 5 | |
| Differentiation of tumour cells | | | |
| Well | 2 | 0 | |
| Moderate | 5 | 3 | N.S.[b] |
| Poor | 2 | 1 | |
| Unclassified | 4 | 2 | |
| Metastasis[c] | | | |
| Presence | 9 | 5 | N.S. |
| Absence | 4 | 1 | |
| Recurrence | | | |
| Presence | 5 | 4 | $P < 0.05$ |
| Absence | 4 | 0 | |
| Not applicable[d] | 4 | 2 | |

[a]Size: $\leqslant 3$ cm = small, $> 3$ cm = large. [b]N.S.: Not significant. [c]Metastasis: regional lymph nodes or liver deposits. [d]Two of these patients died from operative complication and the remaining two had very short follow-up.

from patients with recurrence, while none of the four tumours from the patients without recurrence had allele loss ($P < 0.05$). There was a trend that tumours with poorer differentiation or with metastasis had more allelic losses, but the differences were not statistically significant (Table III).

### Discussion

This study showed loss of heterozygosity on chromosomes 1p33-35 and 11q13 in both exocrine and endocrine carcinomas of the pancreas. Allele loss at 11q13 has been revealed in both sporadic and familial tumours arising in the endocrine pancreas (Bale *et al.*, 1991; Teh *et al.*, 1990). The informative patient with endocrine CaP in our study also had allele loss in this region. Interestingly, there was LOH shown by the marker at this region in two of seven informative cases of exocrine CaP, which has not been reported before. Whether the change in this region is involved in the development of exocrine CaP needs further study.

There are relatively few cytogenetic studies on CaP, but one study of particular interest showed deletion on chromosome 1p32 in one tumour and a translocation involving that breakpoint in a second (Johansson *et al.*, 1991). Allele loss at 1p33-35 was shown by the probe Lambda MS1 in this study in both exocrine (three out of 12 informative cases, Table I) and endocrine (2/2, Table I) CaP, which may indicate a possible tumour suppressor gene located there for both types of CaP, but as this region is frequently involved in advanced cancers of other types, its loss may be related to tumour progression (reviewed in Sager, 1989). More cases are needed to confirm the preliminary finding. It is of interest that allele loss also occurred on chromosome 1q in both endocrine cases, which may suggest that loss of genetic material in this region may be of importance for endocrine tumours.

Recently, loss or mutation of the p53 tumour suppressor gene at 17p13 has been seen at very high frequency in several common human malignancies (Stanbridge, 1990). A recent study in exocrine CaP also showed high frequency of overexpression of mutant forms of p53 by immunohistochemistry and of point mutations of the p53 gene by direct sequencing of genomic DNA (Barton *et al.*, 1991). Hence it was surprising to find that there was no allele loss shown by either probe (p144-D6 or pYNZ22) at 17p13 in either group of CaP in our study. This was in agreement with the finding of Westbrook *et al.* (1990), who did not find any LOH with pYNZ22 in seven informative pancreatic adenocarcinomas. It will be of interest to know if there is any overexpression of mutant p53 or point mutation of the p53 gene in our two groups of CaP.

Frequent rearrangement or loss of the prototype tumour suppressor gene, retinoblastoma (RB), also occurs in some other types of tumours (Horowitz *et al.*, 1990). No allele loss was shown by one of the cDNA probes from the RB gene in the two groups of CaP in this study.

Westbrook *et al.* (1990) reported allele loss in two out of seven informative exocrine CaP on chromosome 5 and suggested that the genetic changes associated with allele loss on that chromosome might be a common denominator in the development or progression of the gastrointestine cancers including those of colorectum and pancreas. In our study, the one informative endocrine CaP showed allele loss at 5q21-22, but four probes on chromosome 5 did not reveal LOH in the exocrine CaP group.

Vogelstein *et al.* (1989) reported that for colorectal carcinomas, patients with more LOH had a considerably worse prognosis than did the other patients. In this study we analysed the possible correlation between frequency of loss of heterozygosity and some clinical parameters within the group of exocrine CaP (Table III). There was a significant correlation found between the frequency of allele loss and the tumour size, and presence or absence of recurrence. The other data in Table III also showed a trend toward more aggressive behaviour in tumours with LOH. However it failed to reach statistical significance. A large study should be conducted in order to confirm the significance of these data.

In conclusion, the study showed LOH on chromosomes 1p33-35 and 11q13 in both exocrine and endocrine CaP. In the group of exocrine CaP, patients with larger tumours, or recurrence may have more allelic losses in their tumours.

We are grateful for the generous support of North East Thames Regional Health Authority, the Gloria Miles Cancer Foundation and Quest Cancer Test. DNA probes were kindly provided by Drs A. Jeffreys, J.A.L. Armour, A.-M. Frischauf, A. Hall, Y. Nakamura (Howard Hughes Medical Institute), S.H. Friend, Dr Higgs, M. Litt and MRC HGMP Resource Centre.

### References

ARMOUR, J.A.L., WONG, Z., WILSON, V., ROYLE, N.J. & JEFFREYS, A.J. (1989). Sequences flanking the repeat arrays of human minisatellites: association with tandem and dispersed repeat elements. *Nucleic Acids Res.*, **17,** 4925–4935.

ARMOUR, J.A.L., POVEY, S., JEREMIAH, S. & JEFFREYS, A.J. (1990). Systematic cloning of human minisatellites from ordered array charomid libraries. *Genomics*, **8,** 501–512.

BALE, A.E., NORTON, J.A., WONG, E.L., FRYBURG, J.S., MATON, P.N., OLDFIELD, E.H., STREETEN, E., AURBACH, G.D., BRANDI, M.L., FRIEDMAN, E., SPIEGEL, A.M., TAGGART, R.T. & MARX, S.J. (1991). Allelic loss on chromosome 11 in hereditary and sporadic tumors related to familial multiple endocrine neoplasia type 1. *Cancer Res.*, **51,** 1154–1157.

BARTON, C.M., STADDON, S.L., HUGHES, C.M., HALL, P.A., O'SULLIVAN, C., KLOPPEL, G., THEIS, B., RUSSELL, R.C.G., NEOPTOLEMOS, J., WILLIAMSON, R.C.N., LANE, D.P. & LEMOINE, N.R. (1991). Abnormalities of the p53 tumour suppressor gene in human pancreatic cancer. *Br. J. Cancer*, **64,** 1076–1082.

BLAND, M. (1987). *An Introduction to Medical Statistics.* Oxford University Press: Oxford.

DEVILEE, P., VAN DEN BROEK, M., KUIPERS-DUKSHOORN, N., KOLLYRI, R., KHAN, P.M., PEARSON, P.L. & CORNELISSE, C.J. (1989). At least four different chromosomal regions are involved in loss of heterozygosity in human breast carcinoma. *Genomics*, **5,** 554–560.

DING, S.-F., HABIB, N.A., DOOLEY, J., WOOD, C., BOWLES, L. & DELHANTY, J.D.A. (1991). Loss of constitutional heterozygosity on chromosome 5q in hepatocellular carcinoma without cirrhosis. *Br. J. Cancer*, **64,** 1083–1087.

FEARON, E.R. CHO, K.R., NICRO, J.M., KERN, S.E., SIMONS, J.W., RUPPERT, J.M., HAMILTON, S.R., PREISINGER, A.C., THOMAS, G., KINZLER, K.W. & VOGELSTEIN, B. (1990). Identification of a chromosome 18q gene that is altered in colorectal cancers. *Science*, **247,** 49–56.

FRIEND, S.H., BERNARDS, R., ROGELJ, S., WEINBERG, R.A., RAPAPORT, J.M., ALBERT, D.M. & DRYJA, T.P. (1986). A human DNA segement with properties of the gene that predisposes to retinoblastoma and osteosarcoma. *Nature*, **323,** 643–646.

FUJIMORI, M., TOKINO, T., HINO, O., KITAGAWA, T., IMAMURA, T., OKAMOTO, E., MITSUNOBU, M., ISHIKAWA, T., NAKAGAMA, H., HARADA, H., YAGURA, M., MATSUBARA, K. & NAKAMURA, Y. (1991). Allelotype study of primary hepatocellular carcinoma. *Cancer Res.*, **51,** 89–93.

GRODEN, J., THILVERIS, A., SAMOWITZ, W., CARLSON, M., GELBERT, L., ALBERTSEN, H., JOSLYN, G., STEVENS, J., SPIRIO, L., ROBERTSON, M., SARGEANT, L., KRAPCHO, K., WOLFF, E., BURT, R., HUGHES, J.P., WARRINGTON, J., MCPHERSON, J., WASMUTH, J., LE PASLIER, D., ABDERRAHIM, H., COHEN, D., LEPPERT, M. & WHITE, R. (1991). Identification and characterization of the familial adenomatous polyposis coli gene. *Cell*, **66,** 589–600.

HIGGS, D.R., WAINSCOAT, J.S., FLINT, J., HILL, A.V.S., THEIN, S.L., NICHOLLS, R.D., TEAL, H., AYYUB, H., PETO, T.E.A., FALUSI, A.G., JARMAN, A.P., CLEGG, J.B. & WEATHERALL, D.J. (1986). Analysis of human adult alpha-globin gene cluster reveals a highly informative genetic locus. *Proc. Natl Acad. Sci. USA*, **83,** 5165–5169.

HOROWITZ, J.M., PARK, S.H., BOGENMANN, E., CHENG, J.-C., YANDELL, D.W., KAYE, F.J., MINNA, J.D., DRYJA, T.P. & WEINBERG, R.A. (1990). Frequent inactivation of the retinoblastoma anti-oncogene is restricted to a subset of human tumor cells. *Proc. Natl Acad. Sci. USA*, **87,** 2775–2279.

JOHANSSON, B., BARDI, G., HEIM, S., MANDAHL, N., ANDREN-SANDBERG, A. & MITELMAN, F. (1991). Cytogenetic analysis of pancreatic adenocarcinomas. *Cancer Genet. Cytogenet.*, **52,** 238–239.

KINZLER, K.W., NILBERT, M.C., SU, L.-K., VOGELSTEIN, B., BRYAN, T.M., LEVY, D.B., SMITH, K.J., PREISINGER, A.C., HEDGE, P., MCKECHNIE, D., FINNIEAR, R., MARKHAM, A., GROFFEN, J., BOGUSKI, M.S., ALTSCHUL, S.F., HORII, A., ANDO, H., MIYOSHI, Y., MIKI, Y., NISHISHO, I. & NAKAMURA, Y. (1991*b*). Identification of FAP locus genes from chromosome 5q21. *Science*, **253,** 661–665.

KINZLER, K.W., NILBERT, M.C., VOGELSTEIN B., BRYAN, T.M., LEVY, D.B., SMITH, K.J., PREISINGER, A.C., HAMILTON, S.R., HEDGE, P., MARKHAN, A., CARLSON, M., JOSLYN, G., GRODEN, J., WHITE, R., MIKI, Y., MIYOSHI, Y., NISHISHO, I. & NAKAMURA, Y. (1991*a*). Identification of a gene located at chromosome 5q21 that is mutated in colorectal cancers. *Science*, **251,** 1366–1370.

KOK, K., OSINGA, J., CARRITT, B., DAVIS, M.B., VAN DER HOUT, A.H., VAN DER VEEN, A.Y., LANDSVATER, R.M., DE LEIJ, L.F.M.H., BERENDSEN, H.H., POSTMUS, P.E., POPPEMA, S. & BUYS, C.H.C.M. (1987). Deletion of a DNA sequence at the chromosomal region 3p21 in all major types of lung cancer. *Nature*, **330,** 578–581.

KONDOLEON, S., VISSING, H., LUO, X.Y., MAGENIS, R.E., KELLOGG, J. & LITT, M. (1987). A hypervariable RFLP on chromosome 17p13 is defined by an arbitrary single copy probe p144-D6 [D17S34]. *Nucleic Acids Res.*, **15,** 10605.

KRONTIRIS, T.G., DIMARTINO, N.A., COLB, M. & PARKINSON, D.R. (1985). Unique allelic restriction fragment of the human Ha-*ras* locus in leukocyte and tumour DNAs of cancer patients. *Nature*, **313,** 369–374.

LARSSON, C., SKOGSEID, B., OBERG, K., NAKAMURA, Y. & NORDENSKJOLD, M. (1988). Multiple endocrine neoplasia type I gene maps to chromosome 11 and is lost in insulinoma. *Nature*, **332,** 85–87.

LEE, W.H., BOOKSTEIN, R., HONG, F., YOUNG, L.-J., SHEN, J.-Y. & LEE, E.Y.H.P. (1987). Human retinoblastoma susceptibility gene: cloning, identification and sequence. *Science*, **235,** 1394–1399.

MICHELASSI, F., ERROI, F., ANGRIMAN, I. & WESTBROOK, C. (1989). Chromosome 5 allele loss in human gastric, ampullary and pancreatic carcinomas. *Ital. J. Surg. Sci.*, **19,** 341–344.

NAKAMURA, Y., BALLARD, L., LEPPERT, M., O'CONNELL, P., LATHROP, G.M., LALOUEL, J.-M. & WHITE, R. (1988*b*). Isolation and mapping of a polymorphic DNA sequence (pYNZ22) on chromosome 17p [D17S30]. *Nucleic Acids Res.*, **16,** 5707.

NAKAMURA, Y., FUJIMOTO, E., O'CONELL, P., LEPPERT, M., LATHROP, G.M., LALOUEL, J.-M. & WHITE, R. (1987). Isolation and maping of a polymorphic DNA sequence pEFD1265.3 on chromosome 9q [D9S7]. *Nucleic Acids Res.*, **15,** 10607.

NAKAMURA, Y., LATHROP, M., LEPPERT, M., DOBBS, M., WASMUTH, J., WOLFF, E., CARLSON, M., FUJIMOTO, E., KRAPCHO, K., SEARS, T., WOODWARD, S., HUGHES, J., BURT, R., GARDNER, E., LALOUEL, J.-M. & WHITE, R. (1988*a*). Localization of the genetic defect in familial adenomatous polyposis within a small region of chromosome 5. *Am. J. Hum. Genet.*, **43,** 638–644.

SAGER, R. (1989). Tumor suppressor genes: the puzzle and the promise. *Science*, **246,** 1406–1412.

SAMBROOK, J., FRITSCH, E.F. & MANIATIS, T. (1989). *Molecular Cloning: a Laboratory Manual.* 2nd ed. Cold Spring Habor Laboratory: New York.

STANBRIDGE, E.J. (1990). Human tumor suppressor genes. *Annu. Rev. Genet.*, **24,** 615–657.

TEH, B.T., HAYWARD, N.K., WILKINSON, S., WOODS, G.M., CAMERON, D. & SHEPHERD, J.J. (1990). Clonal loss of INT-2 alleles in sporadic and familial pancreatic endocrine tumours. *Br. J. Cancer*, **62,** 253–254.

VAN DER STRATEN, A., HERZOG, A., JACOBS, P., CABEZON, T. & BOLLEN, A. (1983). Molecular cloning of human haptoglobin cDNA: evidence for a single mRNA coding for $a^2$ and b chains. *EMBO J.*, **2,** 1003–1007.

VARESCO, L., THOMAS, H.J.W., COTTRELL, S., MURDAY, V., FENNELL, S.J., WILLIAMS, S., SLARLL, S., SHEER, D., BODMER, W.F., FRISCHAUF, A.-M. & SOLOMON, E. (1989). CpG island clones from a deletion encompassing the gene for adenomatous polyposis coli. *Proc. Natl Acad. Sci. USA*, **86,** 10118–10122.

VOGELSTEIN, B., FEARON, E.R., KERN, S.E., HAMILTON, S.R., PREISINGER, A.C., NAKAMURA, Y. & WHITE, R. (1989). Allelotype of colorectal carcinoma. *Science*, **244,** 207–211.

WESTBROOK, C.A., NEUMAN, W.L., JACOBY, R.F., WASYLYSHYN, M., ANGRIMAN, I., ERROI, F. & MICHELASSI, F. (1990). Similar genetic alterations occur in gastric, pancreatic and colorectal carcinomas. *Am. J. Hum. Genet.*, **47,** A24.

WONG, Z., WILSON, V., PATEL, I., POVEY, S. & JEFFREYS, A.J. (1987). Characterization of a panel of highly variable minisatellites cloned from human DNA. *Ann. Hum. Genet.*, **51,** 269–288.